# Rehabilitation Using Implants with Sloped Platform Edge vs. Standard Platform with Guided Bone Regeneration: A Randomized Control Clinical Trial

**DOI:** 10.3390/dj12070205

**Published:** 2024-07-04

**Authors:** Igor Ashurko, Andrey Samsonov, Anna Galyas, Marina Petukhova, Svetlana Tarasenko, Alexey Unkovskiy

**Affiliations:** 1 Department of Oral Surgery of the Institute of Dentistry, I.M. Sechenov First Moscow State Medical University (Sechenov University), 119048 Moscow, Russia; ashurko_i_p@staff.sechenov.ru (I.A.); andsamsonov_rom@mail.ru (A.S.); galyas_a_i@staff.sechenov.ru (A.G.); tarasenko_s_v@staff.sechenov.ru (S.T.); 2 Department of Prosthodontics, Geriatric Dentistry and Craniomandibular Disorders, Charité—Universitätsmedizin Berlin, Aßmannshauser Str. 4–6, 14197 Berlin, Germany

**Keywords:** alveolar bone grafting, implant therapy, dental implantation, bone regeneration, alveolar bone loss, dental implant

## Abstract

The purpose of this study was to evaluate the vertical bone loss after using different techniques: sloped implants or standard implants with guided bone regeneration. Patients with tooth gap and horizontal bone deficiency were randomly allocated to the test group (implants with sloped platform—SLP) and control group (standard design implants with guided bone regeneration—GBR). The primary outcome was bone loss (6 months after finishing the prosthetic treatment). Secondary outcomes included the following: patient-reported outcome measures (PROMs), post-operative edema, keratinized mucosa width, and pink aesthetic score (PES). The average bone loss at 6 months was 0.23 ± 0.15 mm and 1.03 ± 0.37 mm in the SLP and GBR groups, respectively. The SLP group was characterized by lower pain intensity the first 7 days (*p* < 0.001), lower post-operative edema (*p* < 0.001), lower consumption of NSAIDs on days 1, 3, 5, and 7 (*p* = 0.002, <0.001, <0.001, and 0.008), and lower total OHIP-14 (*p* = 0.047) on day 7. The keratinized mucosa width was 3.7 (3.4–4.0) mm and 2 (1.4–2.0) mm in the SLP and GBR groups, respectively. The preservation of the mesial, distal papillae, and the level of soft tissue correspondence were significantly higher in the SLP group (*p* = 0.003, 0.038, <0.001). In the SLP group, more natural color and better texture of soft tissues were found (*p* = 0.048, *p* = 0.041). The use of implants with a sloped platform resulted in superior outcomes compared to the standard-design implants with GBR.

## 1. Introduction

The loss of a tooth due to trauma or extraction inevitably leads to a reduction in the volume of the alveolar ridge. Remodeling processes resulted in a discrepancy in bone height of around 2 mm between the buccal and lingual aspects of the ridge [1]. Such bone atrophy, in some cases, causes the impossibility to place conventional implants without a bone augmentation procedure [2,3].

Dental implantology is actively developing and implementing various methods of bone reconstruction before implant placement. Some of the most commonly used approaches are the use of autogenous bone grafts and the use of guided bone regeneration (GBR).

Despite the availability of a sufficient amount of experimental and clinical data confirming the effectiveness of these methods, it should be noted that they are all associated with certain risks. In particular, the probability of complications, such as dehiscence of postoperative sutures, infection of the surgical wound, and membrane exposure which can reach rates of 5–51% in the case of GBR. Damage to the mandibular canal, paresthesia, bleeding during collection, and transplantation of bone blocks occur in 24.7% of cases [4].

Approaches aimed at minimizing the need for bone augmentation procedures are becoming increasingly relevant. One of them is using the buccal bone as a reference for implant placement, but subcrestal positioning of the implant at the lingual aspect could lead to bone resorption [5].

Another option is the development and implementation of short and ultra-short implants, which avoid bone reconstruction procedures and, as a result, reduce the risk of complications [6,7,8,9]. But there is some evidence that short implants in function for more than 3 years presented higher failure rates compared to regular implants [10,11].

In recent years, implants with a modified design have appeared, one example being implants with a sloped platform. The distinguishing feature of these implants lies in their altered platform configuration: one edge of the implant is positioned lower than the opposite edge, creating a sloping plane. This design enables the implant to be installed when there is a discrepancy in height between the vestibular and oral edges of the bone and follows the bony anatomy. 

According to clinical studies, implants with a sloped platform provide stability to the bone and soft tissues in case of vertical discrepancy in the alveolar ridge. This helps to avoid potential complications during bone augmentation surgery and makes the rehabilitation more accessible and predictable [12,13,14,15]. 

Additionally, successful use of implants with a similar platform design for implantation in edentulous patients using an All-on-4 approach has been described, as it minimizes the reduction in the distal bone in the case of tilted implants [16,17].

Despite the promising clinical outcomes observed in experimental and clinical studies involving implants with a sloped platform, it remains unclear whether these implants offer a more predictable solution than conventional implants accompanied by bone augmentation procedures. The purpose of this study was to evaluate the vertical bone loss after using different techniques: sloped implants or conventional implants with guided bone regeneration.

## 2. Materials and Methods

### 2.1. Study Design

This study was designed as a parallel-arm, randomized, and controlled clinical trial, conducted at the Department of Oral Surgery I.M. Sechenov First Moscow State Medical University (Sechenov University) from September 2021 to December 2023. This study was performed in accordance with the ethical principles of biomedical research formulated in the Declaration of Helsinki of the World Medical Association. Approval for this study (No. 22-21 dated 12 September 2021) was issued by the local ethics committee of Sechenov University, and this trial was also registered in https://clinicaltrials.gov/ with the identifier NCT06404944. All patients signed voluntary informed consent for participation in this study.

The criteria for inclusion in this study were as follows: age over 21 years, loss of tooth in the posterior mandible, the presence of a bone defect H1I according to the Cologne classification of bone defects, an alveolar crest of at least 4.0 mm width and 10 mm height available with sloped design, adjacent teeth without pathologies, an optimal level of oral hygiene, and the absence of general diseases in the acute or decompensation stage [18]. 

Exclusion criteria were as follows: the presence of concomitant diseases in the acute or decompensated stage, patients with a medical history of tumor or irradiation or chemotherapy over the past 5 years, taking drugs that affect bone regeneration, and patients with a history of smoking more than 10 years.

According to the inclusion and exclusion criteria, 30 patients were enrolled in this study. No dropouts were observed during this study.

Prior to the commencement of this study, utilizing spreadsheet software, a list of block randomization was compiled. The randomization process was facilitated with the R 4.3.1 (R Foundation for Statistical Computing, Vienna, Austria) software. The allocation concealment was achieved through the use of sealed envelopes. Both the patient and the operator were blinded prior to surgery. Subsequently, a pre-prepared envelope was opened, and the randomization was executed to ascertain the patient’s assignment to a specific group:

- Group 1 (test group; SLP): implant with a sloped platform edge (*n* = 15);
- Group 2 (control group; GBR): implant with conventional platform + guided bone regeneration (*n* = 15).

### 2.2. Intervention

**Figure 1:**
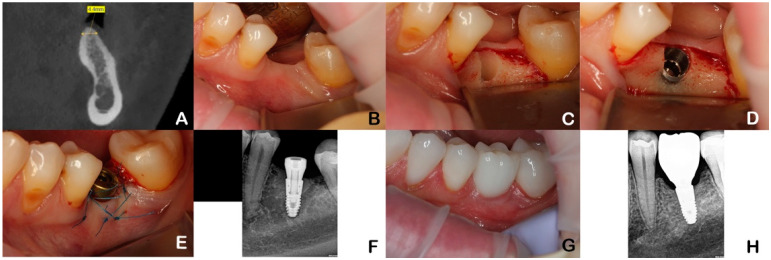
Case illustrating a patient in the test group (SPT). (**A**)—implant planning. (**B**)—single tooth gap. (**C**)—formation of implant site (buccal bone deficiency). (**D**)—implant with sloped platform positioning. (**E**)—suturing around healing abutment. (**F**)—RVG (radiovisiography). (**G**)—6 months post installation of permanent prosthesis. (**H**)—RVG 6-month follow-up.

All patients were examined and operated on by one oral surgeon. After infiltration anesthesia, an incision was made in the middle of the alveolar ridge in the area of the missing tooth, and a full-layer mucosal–periosteal flap was removed. Then, the patients of the test group (SLP) received an implant with a sloped platform edge (Astra Tech Osseospeed EV Profile, Dentsply Sirona, Charlotte, NC, USA) according to the manufacturer protocol and a healing abutment. The mucosal–periosteal flap was sutured with Prolene 6.0 suture material (Johnson & Johnson, New Brunswick, New Jersey, USA) without tension around the healing abutment. After 3 months, the patients were referred to the department of prosthodontics for delivery of the final fixed dental prosthesis (Figure 1).

Patients of the control group (GBR) received an implant of standard design (Astra Tech OsseoSpeed TX implant). GBR was conducted with the standard protocol. After implant placement, vertical incisions were made along the buccal surface in the area of adjacent teeth (or one vertical incision) and a trapezoidal (or L-shaped) mucoperiosteal flap was peeled off. An augmentation procedure was performed in the area of peri-implant dehiscence by combining a Bio-Gide collagen membrane (Geistlich Biomaterials, Wolhusen, Switzerland) with a particulate bone substitute BioOss (Geistlich, Wolhusen, Switzerland). Then, a release incision of the periosteum was performed and the mucoperiosteal flap was returned to its place and sutured with Prolen 6|0 (Johnson & Johnson, New Brunswick, NJ, USA) without tension using U-shaped and simple knotted sutures. After 4 months, the patients underwent the stage of implant opening and a healing abutment was installed. After 2 weeks, the patients were referred to an orthopedic dentist for the manufacture of a prosthetic restoration. Thus, 6 months after the operation, patients in both groups completed the stage of prosthetic treatment.

### 2.3. Clinical Measurements

The second clinician performed the study measurements and was unaware of the intervention type. The primary endpoint of this study was the crestal bone loss around the implant 6 months after finishing the prosthetic treatment. This parameter was determined by analyzing radiovisiography images performed on the Vatech EzSensor device (Vatech, Hwaseong-si, Gyeonggi-do, Republic of Korea) with an X-ray load of 2 μSv. Intraoral radiographs were recorded with the paralleling technique two times with each patient during this study: after implant placement and 6 months after finishing the prosthetic treatment. The radiographs were studied by an examiner using X-ray software (EzDent-I, Vatech, Hwaseong-si, Gyeonggi-do, Republic of Korea) on a 27-inch monitor (ASUS, Beitou District, Taipei, Taiwan) with a screen resolution of 2560 × 1440 pixels with 10× magnification. The measurement of bone loss was based on the calibration with implant diameter as the reference point. Bone loss around dental implants was measured from the top of the implant to the coronal aspect of the alveolar crest on the mesial and distal sides of the implants on radiographs using a digital image analysis program. Bone loss and comparison between groups and within groups was analyzed separately on distal and mesial implants. 

Secondary endpoints were the following: the Pink Esthetic Score (PES), the Visual Analogue Scale (VAS), number of analgesic medications taken, post-operative edema/swelling, duration of surgery, implant stability (ISQ), width of keratinized mucosa, and the oral health impact profile (OHIP-14).

The PES table was used to evaluate aesthetic parameters 1 month after finishing the prosthetic treatment [19]. To assess the condition of the soft tissues, a reference tooth (adjacent or on the contralateral side) was selected. The implant area and the reference tooth were digitally photographed using a Canon EOS450D camera (Canon Inc., Tokyo, Japan) equipped with a Canon Macro Ring Lite MR-14EX (Canon Inc., Japan) circular flash. The images were magnified twofold from their original size. On a scale of zero to two, the following parameters were evaluated: the preservation of the medial and distal papillae, soft tissue level mismatch, soft tissue contour mismatch, alveolar ridge deficiency, soft tissue color, and soft tissue texture, where the score “0” denoted an unsatisfactory result and “2” denoted an aesthetic result close to natural.

A ten-point VAS scale was used to assess postoperative pain, where “0” denoted the absence of pain and “10” denoted intolerable pain, which was self-completed by the patient on days 1, 3, 5, 7, 90, and 180.

The number of analgesic medications (Nimesulide (100 mg)) taken by the patient was estimated according to the table, which patients filled in independently on the 1st, 3rd, 5th, and 7th day after surgery.

Post-operative edema/swelling was evaluated with visual assessment on days 1, 3, 5, and 7 using the following scoring system: 0 = no visible edema; 1 = slight edema (intra-oral swelling in the surgical zone); 2 = moderate edema (extra-oral swelling in the surgical area); 3 = severe edema (extra-oral swelling extending from the surgical site) and/or visible hematoma and ecchymosis [20].

The operative time was measured using chronometry from the first incision to the last suture.

The implant stability (ISQ) was measured using a Penguin device (Integration Diagnostics Sweden AB, Göteborg, Sweden) at the time of implant placement and at 180 days.

The width of the keratinized mucosa was measured using the UNC-15 periodontal probe at the buccal side before surgery and 1 month after finishing the prosthetic treatment.

The oral health impact profile questionnaire (OHIP-14) was handed out to patients and filled out at baseline and follow-up at 7, 120, and 180 days. The questionnaire consists of 14 questions about the state of life, health, and subjective feelings of the patient, where the score “0” noted the absence of a problem and “4” noted a high concern of the patient about the problem.

### 2.4. Statistical Analyses

Statistical analyses and visualization of the obtained data were carried out using the environment for statistical computing R 4.3.1 (R Foundation for Statistical Computing, Vienna, Austria).

The sample size for this study was calculated to detect a difference in the rate of bone resorption at the implant neck of at least 0.3 mm between the two groups (standard deviation [SD] 0.2 mm and mean 0.6 + −0.2 mm), which was adapted from an article published by Meloni S. M. et al., 2018. The number of patients in each group was calculated to be 14 (alpha = 0.05; power = 80%). This number was increased by 5% to account for possible exclusions from this study. Thus, it was planned to include 30 patients in this study (15 in each group).

The Shapiro–Wilk test (goodness of fit) was conducted to indicate the data distribution. Descriptive statistics for categorical variables are presented in the form of absolute and relative frequencies for quantitative variables in the form of medians (1st–3rd quartiles). The Fisher test was used to compare groups with respect to categorical variables. The Mann–Whitney test was used to compare the two groups with respect to quantitative and ordinal indicators. Mixed models of proportional odds with the inclusion of the interaction term between the observation period and the group indicator were used to compare the dynamics of changes in quantitative and ordinal indicators. The differences were considered statistically significant at *p* < 0.05. Statistical analysis was conducted by the blinded investigator.

## 3. Results

This study included 23 women and 7 men aged from 24 to 64 years. A total of 150 patients with a single tooth gap (ICD-10: K08.1—loss of teeth due to an accident, extraction, or local periodontal disease) were screened (Table 1).

There were no statistically significant differences between the patient groups with respect to the height (*p* = 0.44) and the width (*p* = 0.061) of the bone ridge before implantation. The crestal bone loss relative to the implant platform 6 months after installing final restoration, both mesial and distal, was more severe in the GBR group, and the average amounted to 0.23 ± 0.15 mm and 1.03 ± 0.37 mm in the SLP and GBR groups, respectively (*p* < 0.001) (Table 2).

The Pink Esthetic Score (PES) revealed that the preservation of the medial and distal papillae was statistically significantly higher in SLP group compared with GBR group (*p* = 0.001). The tissue level mismatch was highest in the SLP group compared with the GBR group (*p* < 0.001). Soft tissue contour naturalness was not statistically significant (*p* = 0.011). There were no statistically significant differences between the groups in the assessment of alveolar ridge deficiency (*p* = 0.886). When assessing the color of soft tissues in patients in the SLP group, a more natural color was noted compared with the GBR group (*p* = 0.003); patients in the SLP group also tended to have a better texture of soft tissues (*p* = 0.041) (Table 3).

Patients in the GBR group noted more pronounced postoperative pain on days 1, 3, 5, and 7 after surgery (*p* < 0.001). There were no differences between the groups 3 and 6 months after surgery (Table 4).

More pronounced postoperative pain in the GBR group is also confirmed by a large amount of analgesic medication taken on the 1st, 3rd, 5th, and 7th days after surgery (Table 5).

**Figure 2:**
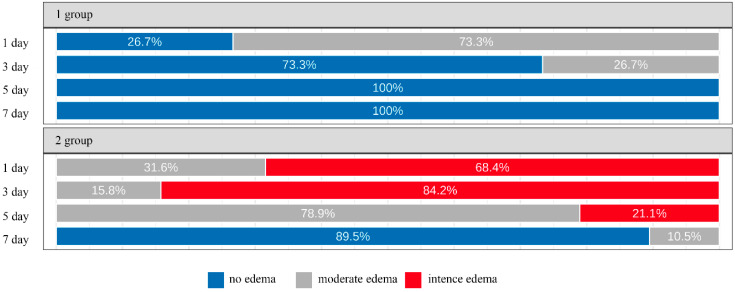
Dynamic of the severity of post-operative edema after surgery (%).

The post-operative edema/swelling of soft tissues on the 1, 3 and 5 day after surgery was statistically significantly higher in GBR group (*p* < 0.001) (Figure 2).

The duration of intervention in the SLP group was 31 (29–34.5) minutes and in the GBR group was 87 (75.5–101) minutes, the difference between groups was 56 min [95% CI: 42; 60] (*p* < 0.001).

The Shapiro–Wilk test revealed the normal distribution of the data. There was a statistically significant difference between the patients in both groups in the implant stability (ISQ) at the time of implant placement and 4 months after the surgery. Thus, at the time of implant placement, the ISQ was 73 (71–77) and 62 (58.5–67.5) in the SLP group and GBR group, respectively. At 4 months after implant placement, the ISQ was 85 (81–86.5) and 71 (70.5–74.5) in the SLP group and GBR group, respectively.

The width of the keratinized mucosa at the buccal site 1 month after finishing the prosthetic treatment was statistically significantly higher in the SLP group compared with the GBR group and amounted to 3.7 (3.4–4.0) mm and 2 (1.4–2.0) mm, respectively.

At 7 days after surgery, the SLP group had a statistically significantly lower total OHIP-14 score compared with the GBR group (*p* = 0.001). At 4 months after surgery, there were no statistically significant differences between patients regarding the total OHIP-14 score (*p* = 0.743). At 6 months after surgery, there was a trend towards a lower total OHIP-14 score in group 1 (*p* = 0.056) (Table 6).

## 4. Discussion

One of the factors affecting the prognosis of implant function is the surrounding bone volume. Atrophy which occurs after tooth extraction often prevents implant placement without additional bone grafting procedures. At the same time, bone augmentation increases the rehabilitation time and the risk of complications [21].

One of the methods to avoid bone grafting procedures is the use of implants with a sloped platform [12,13]. This article describes a comparative analysis of the use of an implant with a sloped platform and a conventional implant with guided bone regeneration.

According to the results of this study, all implants in both groups were successfully integrated. Compared with another recently published study, published by Schiegnitz et al., the presented results indicated a high survival rate for the sloped implant [12].

It was noted that the implant stability (ISQ) at the time of surgery in the SLP group was statistically significantly higher than in the GBR group. This is probably explained by the fact that in patients of GBR group, due to atrophy of the alveolar ridge, the implant was not completely surrounded by bone. By the time of prosthetics, there was a tendency to increase ISQ in patients of both groups.

The average crestal bone loss for the implant with a sloped platform edge in our study was slightly less than in studies conducted by Schiegnitz et al. (0.3 mm), Noelken et al. (0.54 mm), and Donati et. al. (0.57 mm) [12,13,22]. At the same time, Schiegnitz et al. and Donati et. al. published the long-term results (20.7 ± 8 months and 3 years, respectively). Donati et. al. showed marginal bone loss of about 0.57 mm during the 3-year period with sloped implants. While the average bone loss between 1 and 3 years amounted to 0.30 mm, approximately 50% of the implants showed no bone loss during this period.

In the present study, assessment of crestal bone loss was performed only 6 months after installing the final restoration, which is a limitation of this study, as additional bone loss may occur thereafter, and more distant results are needed.

It should be noted that the magnitude of bone resorption depends on many factors, including the method of surgery. For example, in a prospective study by Noelken et al., marginal bone loss on tilted implants at 3-year follow-up after immediate implant placement and provisionalization averaged to 0.2 mm [23]. Puisys et al. compared marginal bone loss for sloped and conventional implants in edentulous patients using an All-on-4 approach. Data obtained 1 year after implant placement demonstrate that sloped (0.29 mm) and conventional (0.22 mm) implants have the same potential for minimal crestal bone loss [16]. These results showed stable marginal bone for implants with a sloped configuration.

In the present trial, the crestal bone loss around regular implants with GBR was more severe than that found around sloped implants. This correlates with other studies [22,24]. It was noted that the crestal bone loss in guided bone regeneration with implant installation can continue from month 6 to month 9 after prosthesis delivery [24]. This suggests that, in the present study, the difference between both groups may be even more pronounced in the long-term follow-up period.

It is important to note that the current study assessed interproximal marginal bone level changes based on contact radiography. The utilization of computed tomography could have enabled the evaluation of bone loss levels both vestibularly and orally, which would have been valuable information. However, firstly, the use of control CT scans is contraindicated due to ethical reasons regarding radiation reduction. Secondly, the background created by the metal may impede accurate assessment of the bone level.

The results of the PES index also correlate with the results of similar studies in which the authors found lower aesthetic scores in patients after bone grafting compared to implant placement in conditions of sufficient bone volume [25]. In addition, in a study by De Bruyckere et al., the authors additionally investigated the Mucosal Scarring Index (MSI), which was significantly higher in patients in the group with GBR [25,26].

The decrease in the width of the keratinized mucosa in the postoperative period in the GBR group is due to additional incisions for mobilization of the mucosal periosteal flap, which is a necessity, when performing osteoplastic operations. Apparently, this is also associated with a less aesthetic result of treatment in patients in this group after completion of prosthetics, since such operations are always associated with the loss of attached mucosa.

On the other hand, the SLP group exhibited a slight increase in the width of keratinized mucosa one month finishing the prosthetic treatment compared to the initial state. It is important to note that the measurement of keratinized mucosa width in this study was conducted using a standard UNC-15 probe with a graduation of 1 mm, which is not an entirely precise method of assessment and may represent a limitation of the current investigation. Nevertheless, a similar and statistically significant increase in peri-implant keratinized mucosa width over time in the area of implants with a sloped platform has also been described in the study by Schiegnitz et al. [12].

According to the present study, the severity of pain and post-operative edema were statistically significantly higher in patients who underwent GBR, which directly affected the patients’ quality of life. These findings are consistent with the results of a study by De Bruyckere et al., who has also noted the formation of hematomas and more pronounced collateral edema on days 1, 3, and 7 after surgery in patients after guided bone regeneration compared with patients with only soft tissue augmentation during implantation [25].

### Limitations

Some limitations exist with respect to the present clinical study. This investigation was based on a relatively small sample size, which may have impacted the results of the statistical comparisons between the groups. Furthermore, this study is characterized by a brief period of observation. To ascertain the clinical efficacy of using implants with a sloped platform, more long-term follow-ups are imperative. We intend to conduct extended monitoring of these patients for a minimum of three years. Additionally, one of the constraints is the absence of an assessment of peri-implant health-related parameters. Another limitation of the present study could be due to significant difference (*p* = 0.001) in the implant diameters used. There are some data that suggest an increase in the implant diameter produces significant stress reduction in the alveolar crest [27]. It is necessary to take this factor into account in further studies.

## 5. Conclusions

The use of implants with a sloped platform resulted in superior outcomes compared to standard design implants with guided bone regeneration. This approach is characterized by a reduction in surgical intervention time, a decrease in postoperative discomfort, and a satisfactory aesthetic result. Therefore, it can be used as an alternative for rehabilitation of patients with horizontal atrophy of the alveolar ridge. Further studies are warranted to evaluate clinical outcomes in the long-term period.

## Figures and Tables

**Table 1:** Baseline characteristics.

Characteristic	All Patients	SLP	GBR	*p*-Value
**Age (years)**	42.2 (±10.8) 40 (34–47)	41.5 (±8.9) 40 (34–45)	43.5 (±13.9) 39 (32–57)	0.547
**Gender**				0.076
Female	23 (76.7%)	11 (73.3%)	12 (80.0%)	
Male	7 (23.3%)	4 (26.7%)	3 (20.0%)	
**Implant position**				0.175
First molar	27 (90%)	14 (93.3%)	13 (86.7%)	
Second molar	3 (10%)	1 (6.7%)	2 (13.3%)	
**Implant length (mm)**				0.08
8	4 (6.6%)	2 (13.3%)	2 (13.3%)	
9	22 (73.3%)	10 (66.7%)	12 (80%)	
11	4 (13.3%)	3 (20%)	1 (6.6%)	
**Implant diameter (mm)**				**0.001**
4.0	6 (20%)	0 (0%)	6 (40%)	
4.3	15 (50%)	15 (100%)	0 (0%)	
4.5	5 (16.7%)	0 (0%)	5 (33.3%)	
5.0	4 (13.3%)	0 (0%)	4 (26.7%)	
Keratinized mucosa width (mm)		3.1 (2.4–3.2)	2.9 (2.5–3.1)	>0.999

**Table 2:** Results of assessing the width and height of the bone before surgery. Assessment of crestal bone loss 6 months after installing the final restoration.

Characteristic	SLP M (±SD) Me (Q1–Q3)	GBR M (±SD) Me (Q1–Q3)	*p*-Value
Bone ridge height before surgery (mm)	12.7 (±1.7) 12.1 (11.3–14.3)	12.4 (±2.4) 12 (10.1–14.3)	0.44
Bone ridge width before surgery (mm)	4.7 (±0.6) 4.8 (4.3–4.9)	5.5 (±0.9) 5.8 (4.9–6.2)	0.061
Crestal bone loss mesially (mm)	0.21 (±0.14) 0.19 (0.00–0.38)	1.06 (±0.35) 1.10 (1.00–1.20)	**<0.001**
Crestal bone loss distally (mm)	0.25 (±0.15) 0.22 (0.00–0.39)	1.00 (±0.39) 0.90 (0.80–1.20)	**<0.001**
Crestal bone loss average (mm)	0.23 (±0.15) 0.21 (0.00–0.39)	1.03 (±0.37) 1.1 (0.90–1.20)	**<0.001**

**Table 3:** Results of the Pink Esthetic Score (PES) 1 month after installation of the prosthetic structure.

Characteristic	SLP	GBR	*p*-Value
Mesial papilla			**<0.001**
absent	0 (0%)	3 (15.8%)	
incomplete	4 (26.7%)	13 (68.4%)	
complete	11 (73.3%)	3 (15.8%)	
Distal papilla			**<0.001**
absent	0 (0%)	2 (10.5%)	
incomplete	6 (40%)	14 (73.7%)	
complete	9 (60%)	3 (15.8%)	
Tissue level			**<0.001**
discrepancy of more than 2 mm	1 (6.7%)	1 (5.3%)	
1–2 mm discrepancy	1 (6.7%)	13 (68.4%)	
no discrepancy or <1 mm	13 (86.7%)	5 (26.3%)	
Soft tissue contour			**0.011**
not natural	0 (0%)	3 (15.8%)	
quite natural	9 (60%)	14 (73.7%)	
natural	6 (40%)	2 (10.5%)	
Alveolar ridge deficiency			0.886
obvious	0 (0%)	1 (5.3%)	
insignificant	10 (66.7%)	10 (52.6%)	
absent	5 (33.3%)	8 (42.1%)	
Soft tissue color			**0.003**
obvious difference	0 (0%)	0 (0%)	
moderate difference	0 (0%)	6 (31,6%)	
no difference	15 (100%)	13 (68,4%)	
Soft tissue texture			**0.041**
obvious difference	1 (6.7%)	0 (0%)	
moderate difference	1 (6.7%)	7 (36.8%)	
no difference	13 (86.7%)	12 (63.2%)	
Mean (SD)	11.86	8.61	

**Table 4:** Dynamics of postoperative pain severity.

VAS	SLP	GBR	*p*-Value
1 day	2 (1.5–2)	4 (3–5)	**<0.001**
3 days	1 (1–1)	3 (2–4)	**<0.001**
5 days	0 (0–0.5)	1 (1–2)	**<0.001**
7 days	0 (0–0)	1 (0–1)	**<0.001**
90 days			0.085
180 days			0.2

**Table 5:** Dynamics of the amount of NSAID painkillers taken (Nimesulide, 100 mg).

Characteristic	SLP	GBR	*p*-Value
1 day	1 (1–2)	2 (2–3)	**<0.001**
3 days	1 (0–1)	2 (2–3)	**<0.001**
5 days	0 (0–0)	1 (1–2)	**<0.001**
7 days	0 (0–0)	0 (0–1)	**0.002**

**Table 6:** Dynamics of assessing the quality of life of patients (OHIP-14).

Total Score OHIP-14	SLP	GBR	*p*-Value
before surgery	4.8 (±5.1) 5 (0–6.5)	4.7 (±2.5) 4 (3–6.5)	0.807
7 days	8,1 (±4.8) 8 (4.5–11)	13.6 (±4.7) 12 (11–14)	**<0.001**
120 days	1.9 (±1.7) 2 (1–2)	4.5 (±8) 1 (0–5.5)	0.743
180 days	0 (±0) 0 (0–0)	1.5 (±3.4) 0 (0–1)	0.056

## Data Availability

Data is available on reasonable request.
